# Supplementary material for: The ABA/LANCL1/2 Hormone/Receptor System Controls Adipocyte Browning and Energy Expenditure
Source: Int J Mol Sci. 2023 Feb 9;24(4):3489. doi: 10.3390/ijms24043489 (PMC9966313; doi:10.3390/ijms24043489)
Supplement: Supplementary file 1 [file ijms-24-03489-s001.zip › ijms-2176034-supplementary.pdf]

Supplementary data

Table S1. Primer sequences used to amplify mouse target genes.

Mouse genes	Accession N.	Forward Primer 5′-3′	Reverse Primer 5′-3′
Hprt1	NM_013556	CCCTGGTTAAGCATACAGCCCC	AGTCTGGCCTGTATCCAACACTTCG
Lancl1	NM_001190985	GAGGGCCTTTCCGAATCCTT	GGAGTCAGCCTCCCAGTAGA
MT-DNA	AP014941	CCGTCACCCTCCTCAAATTA	GGGCTAGGATTAGTTCAGAGTG
ND1	AP014941	CCACGCTTCCGTTACGATCA	GTATGGTGGTACTCCCGCTG
Adrb3	NM_013462	TCCCAGCGGAGTTTTCATCG	GCTACACAGAAGACGACGGA
Thra	NM_001313983	ACCACCGCAAACACAACATT	TCGAACTCTGCACTTCTCTCTC
Thrb	NM_001113417	ATCTCAAGTGCCCAGACTTTCC	TTTGTCCCCACACACTACACA
Estrra	U85259	CCCTGACAGTCCAAAGGGTT	CATCCTCCTCCTCCTTGTGC

Table S2. Primer sequences used to amplify human target genes.

Human genes	Accession N°	Forward Primer 5′-3′	Reverse Primer 5′-3′
Ucp1	NM_021833	GTGTGCCCAACTGTGCAATG	GTTCCAGGATCCAAGTCGCA
Ucp3	NM_022803	ATACAGCGGGACTATGGACG	ACCTCAGCACAGTTGACGAT
ESRRA	NM_004451	TATGGTGTGGCATCCTGTGAG	GGACAGCTGTACTCGATGCT
DIO2	AF188709	CTCTATGACTCGGTCATTCTGC	TGTCACCTCCTTCTGTACTGG
Slc2a4	NM_001042	AATGCTGCTGCCTCCTATG	ATCAGAATGCCGATAACAATGG
PPARG	NM_138712	CGAAGACATTCCATTCACAAG	CTCCACAGACACGACATTC
CPT1B	NM_004377	AAACAGTGCCAGGCGGTC	CGTCTGCCAACGCCTTG
CIDEA	NM_001279	AGGCAGGTTCACGTGTGGATA	GAAACACAGTGTTTGGCTCAAGA
MT-DNA	AY665667	CCCACCCAAGAACAGGGTTT	TAGGAGGTTGGCCATGGGT

MPC1	NM_001376568	CCTCGGAACTGGCTTCTGTT	CTCGTGTTTGATAAGCCGCC
PDHA1	NM_001173456	ATGGATATCCTGTGCGTCCG	GTGTCCGTGGTAACGGTAAGT
ADRB3	NM_000025	CCTCCAACATGCCCTACGTG	CGTAGACGAAGAGCATCACGA
c-erbA-1	X55005	ATGACACGGAAGTGGCTCTG	AGGTACGCCTCCTGACTCTT
THRB	NM_000461	AGCCACTGGAAGCAAAAACG	CTTTCCACCTTCTGGGGCAT
INSR	AH002851	CTGGGTTTCAAGCGGAGCTA	AAGGATTGGACCGAGGCAAG
Prkaa1	NM_006251	TACATTCTGGGTGACACGCT	AGGCTCCGAATCTTCTGTCG
Sirt1	AF083106	AGGCCACGGATAGGTCCATA	GTGGAGGTATTGTTTCCGGC
Ppargc1a	NM_001330751	CTGTGTCACCACCCAAATCCTTAT	TGTTCGAGAAAAGGACCTTGA
Lancl1	NM_006055	TCTCACAACGCTTGACCAATAAG	GCCCAGCCAGTGTAACCG
Lancl2	AF353942	AGGCGTACAAGGTCTTTAAGGAG	GCTCGGTAGAGGTACTTCTTATCC
ANT1	J04982	TGGTCTGGGCGACTGTATCA	ACTCCGAAGTAGGCAGCTCT
Hprt1	NM_000194	GGTCAGGCAGTATAATCCAAAG	TTCATTATAGTCAAGGGCATATCC
ND1	AY665667	CGATTCCGCTACGACCAACT	GTTTGAGGGGGAATGCTGGA
Lancl3	NM_198511	GGCGAGACCATCGAGAGAGA	GTGTCCAGGTACTGCGGTTT

Table S3. Primary and secondary antibodies used for Western blot.

**Primary Antibody**	**Host**	**Concentrations**	**Manufacturer**
Anti-LANCL1	Rabbit	1:250	Novus Biologicals
Anti-LANCL2	Mouse	1:1000	Reference [31]

Anti-AMPK tot	Rabbit	1:1000	Cell Signaling Technology, Danvers, MA
Anti-pAMPK P-Ser473	Rabbit	1:1000	Cell Signaling Technology, Danvers, MA
Anti-vinculin	Rabbit	1:1000	Cell Signaling Technology, Danvers, MA
Anti-PGC-1α	Mouse	1:1000	Sigma-Aldrich

**Secondary Antibody**	**Concentrations**	**Manufacturer**
Anti-Mouse	1:2000	Santa Cruz Biotechnology Inc., California
Anti-Rabbit	1:1000	Santa Cruz Biotechnology Inc., California

Table S4. Differentiation protocol of human immortalized preadipocytes. To induce adipocyte differentiation TERT-hWA and TERT-hBA human preadipocytes were cultured in advanced DMEM/F12 containing 2% FBS, 2 mM L-glutamine, 62.5 μg/ml penicillin, 100 μg/ml streptomycin (basal medium). The following supplements were added to the basal medium with the indicated timing: insulin (5 μg/ml), dexamethasone (1 μM), 3-isobutyl-1-methylxanthine (IBMX) (0.5 mM), rosiglitazone (1 μM), human cortisol (1 μM) and T3 (1 nM). For white and brown adipocytes, culture stops at day 12. Browning of white to "beige" adipocytes is induced by culture with rosiglitazone between days 12 and 15.

	**TERT-hWA**		**TERT-hBA**
	**white**	**"beige"**	**brown**
Days 0-5	basal medium **+** insulin, dexamethasone, IBMX, rosiglitazone, cortisol, T3		
Days 6-12	basal medium		basal medium + T3
Days 12-15	-	basal medium+ rosiglitazone	-

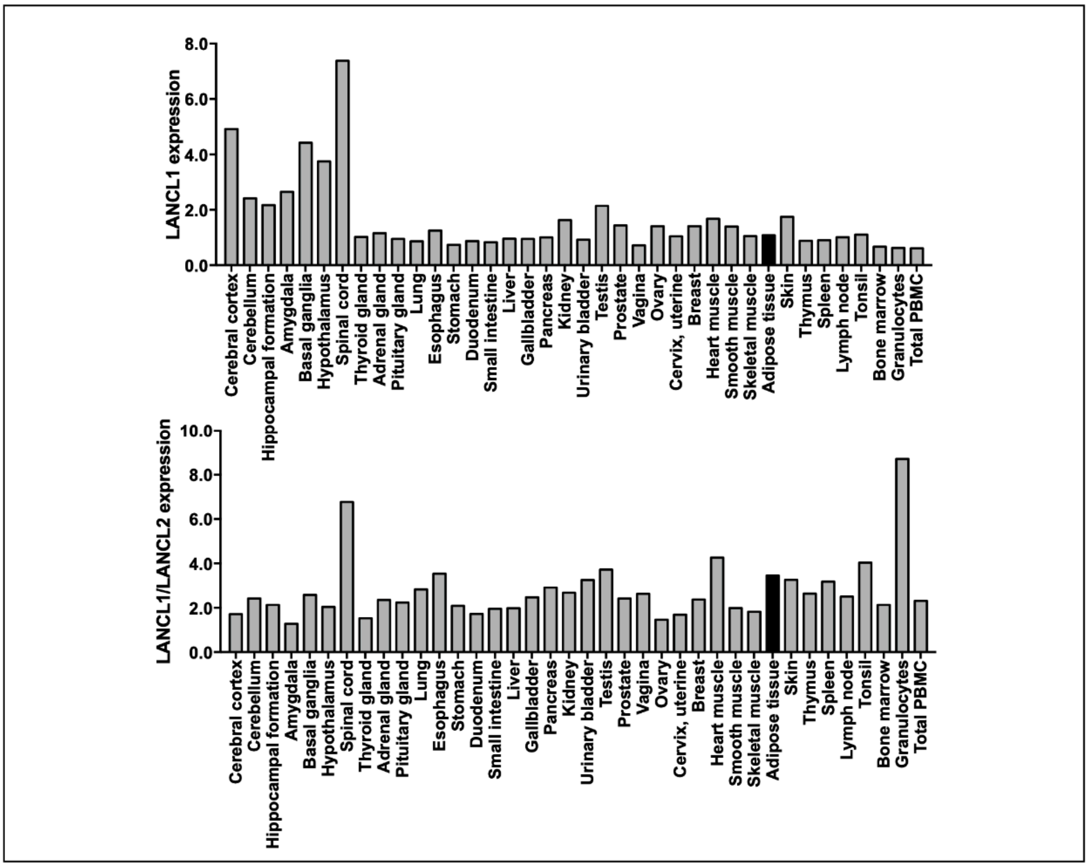

Figure S1. Tissue expression levels of LANCL1 and LANCL2 proteins in human tissues. The data reported are from The Human Protein Atlas portal. LANCL1 expression levels in various tissues relative to the pancreas (upper panel) and LANCL1/LANCL2 expression (lower panel) in different tissues. The mRNA expression levels in human tissue are based on RNA-seq data generated by the Human Protein Atlas, Genotype-Tissue Expression portal and CAGE data generated by the FANTOM5 consortium. Consensus normalized expression levels for human tissue is created by combining the data from these three transcriptomics datasets. Adipose tissue (AT) is indicated by a black bar; in these tissues, LANCL1 expression levels are 3-fold higher than LANCL2 expression levels.

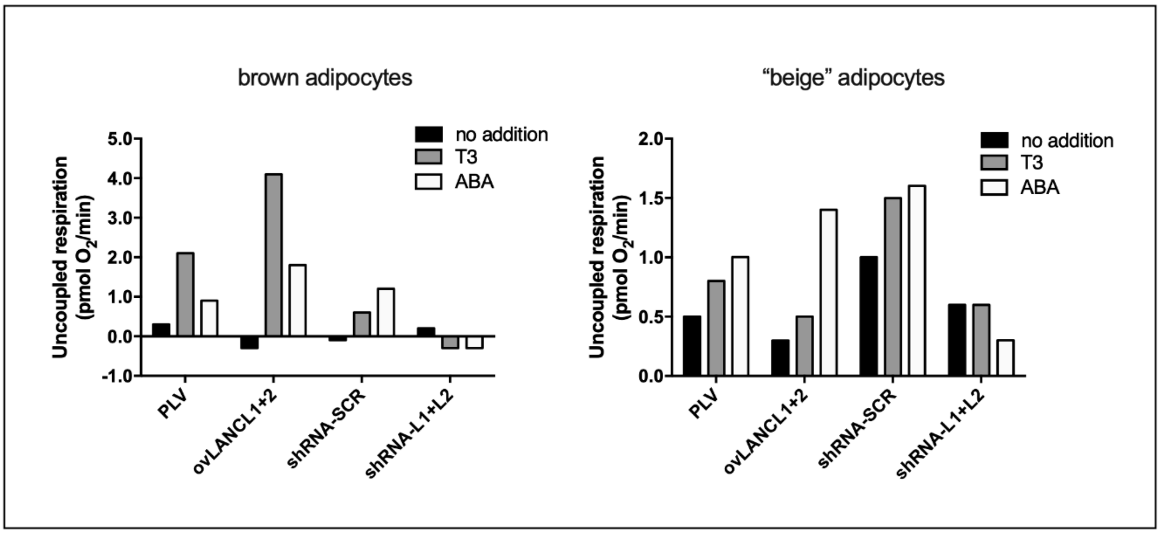

Figure S2. Uncoupled respiration in LANCL1/2-overexpressing and in double-silenced brown and "beige" adipocytes. Uncoupled respiration was calculated as the difference between respiration in the presence of oligomycin and after addition of rotenone/antimycin A. The mean values shown are expressed as pmol O_2/min/5,000 cells and are derived from the data shown in Figure 6.
